# Dengue occurrence relations and serology: cross-sectional analysis of results from the Guerrero State, Mexico, baseline for a cluster-randomised controlled trial of community mobilisation for dengue prevention

**DOI:** 10.1186/s12889-017-4291-y

**Published:** 2017-05-30

**Authors:** Elizabeth Nava-Aguilera, Arcadio Morales-Pérez, Alejandro Balanzar-Martínez, Ofelia Rodríguez-Ramírez, Abel Jiménez-Alejo, Miguel Flores-Moreno, David Gasga-Salinas, José Legorreta-Soberanis, Sergio Paredes-Solís, Pedro Antonio Morales-Nava, María de Lourdes Soto-Ríos, Robert J Ledogar, Joséfina Coloma, Eva Harris, Neil Andersson

**Affiliations:** 1 Centro de Investigación de Enfermedades Tropicales (CIET), Universidad Autónoma de Guerrero, Acapulco, Guerrero Mexico; 2 Facultad de Medicina, Universidad Autónoma de Guerrero, Taxco, Guerrero Mexico; 3 Escuela de Enfermería Número 4, Universidad Autónoma de Guerrero, Taxco, Guerrero Mexico; 4 CIETinternational, New York, USA; 5 Division of Infectious Diseases and Vaccinology, School of Public Health, University of California, Berkeley, CA USA; 6 Department of Family Medicine, McGill University, Montreal, Canada

**Keywords:** Dengue, Infection, Serology, Risk factors

## Abstract

**Background:**

The Mexican arm of the Camino Verde trial of community mobilisation for dengue prevention covered three coastal regions of Guerrero state: Acapulco, Costa Grande and Costa Chica. A baseline cross-sectional survey provided data for community mobilisation and for adapting the intervention design to concrete conditions in the intervention areas.

**Methods:**

Trained field teams constructed community profiles in randomly selected clusters, based on observation and key informant interviews. In each household they carried out an entomological inspection of water containers, collected information on socio-demographic variables and cases of dengue illness among household members in the last year, and gathered paired saliva samples from children aged 3–9 years, which were subjected to ELISA testing to detect recent dengue infection. We examined associations with dengue illness and recent dengue infection in bivariate and then multivariate analysis.

**Results:**

In 70/90 clusters, key informants were unable to identify any organized community groups. Some 1.9% (1029/55,723) of the household population reported dengue illness in the past year, with a higher rate in Acapulco region. Among children 3–9 years old, 6.1% (392/6382) had serological evidence of recent dengue infection. In all three regions, household use of anti-mosquito products, household heads working, and households having less than 5 members were associated with self-reported dengue illness. In Acapulco region, people aged less than 25 years, those with a more educated household head and those from urban sites were also more likely to report dengue illness, while in Costa Chica and Costa Grande, females were more likely to report dengue illness. Among children aged 3–9 years, those aged 3–4 years and those living in Acapulco were more likely to have evidence of recent dengue infection.

**Conclusions:**

The evidence from the baseline survey provided important support for the design and implementation of the trial intervention. The weakness of community leadership and the relatively low rates of self-reported dengue illness were challenges that the Mexican intervention team had to overcome. The higher dengue illness occurrence among women in Costa Grande and Costa Chica may help explain why women participated more than men in activities during the Camino Verde trial.

## Background

Throughout the first decade of the twenty-first century, dengue fever (DF) was a major public health concern. In Mexico as a whole, DF rates increased almost tenfold from 5018 registered cases in 2003, to 48,456 cases in 2007 [1], with a similar trend in the Mexican state of Guerrero. In 2012, Guerrero registered one of the highest DF incidence rates in the country (113 per 100,000), and the fourth highest rate for dengue hemorrhagic fever (DHF) [1]. More recently, dengue has continued to be a public health concern. In 2015, 26,665 cases of DF were reported in Mexico. Of these, 5464 were cases of DHF which resulted in 42 deaths [2]. Guerrero, which comprises 2.9% of the total national population [3], reported 6.5% of DF cases, 9.5% of DHF cases, and one out of four dengue-related fatalities in the country.

Most dengue infections are asymptomatic or present as a non-specific feverish illness, with asymptomatic infections predominating [4–6]. Authors have linked the spread of DF in the Americas to demographic, environmental and biological factors; social changes; the presence of *Aedes aegypti* breeding sites; and deficient public service infrastructure [7–9]. A task as complex as decreasing the spread of dengue requires an integrated approach, with involvement of households and communities as well as public services [10, 11]. The Camino Verde cluster randomised controlled trial (CRCT) in Mexico and Nicaragua (2010–2012) aimed to test the added value of community mobilisation for dengue prevention [12, 13].

This article reports on findings from the 2010 baseline survey for the Mexican arm of the Camino Verde trial, which took place in the three coastal regions of Guerrero State [12, 13]. The baseline surveys in Mexico and Nicaragua were not carried out to serve as points of reference for measuring impact of the interventions. Rather, they provided important data used at the beginning of the trial and during the intervention: for stratifying the random allocation of the sample into intervention and reference communities; for input into the evidence-based community mobilisation; and for adapting the intervention implementation to local conditions in the trial communities. The baseline findings reported here are also useful for interpreting the Camino Verde trial results and evaluating their implications for future action against dengue in Guerrero state, in Mexico as a whole, and elsewhere.

## Methods

The methods of the Camino Verde trial, including the conduct of the baseline surveys, are described in detail elsewhere [12, 13]. A brief description follows here. The Mexican arm of trial took place in a random sample of 90 clusters in the three coastal regions of Guerrero State (Acapulco, Costa Grande and Costa Chica) drawn from the State Health Secretariat’s register of census enumeration areas. Each cluster comprised on average 137 contiguous households. The baseline survey included: a household questionnaire covering demographics and socio-economic status, and self-reported dengue illness in family members during the last 12 months; an entomological survey in the same households examining water containers for *Aedes aegypti* larvae and pupae and the presence of temephos (a larvicidal chemical added to household water containers as a key part of the government vector control programme for dengue prevention); paired saliva samples from children aged 3–9 years in the households to test for dengue-specific IgG antibodies at the beginning and end of the three-month dengue season; and interviews with key informants (anyone with a good knowledge about the community) in each cluster which supported construction of a community profile. The survey instruments drew on experience from a pilot study in Nicaragua [14], and trained local fieldworkers carried out the interviews and entomological inspections, and obtained the saliva samples. They obtained consent to work in the communities from local leaders and sought oral informed consent in each household.

### Measurement of dengue antibodies

We used an ELISA procedure for detecting dengue-specific IgG antibodies in the paired saliva samples [15, 16]. We used dedicated software for automatic optical density transfer from the ELISA reader to the computer, and used the same programme to estimate IgG units, to estimate if there was recent dengue infection or not, and to check for data concordance [17].

### Data management and analysis

Trained operators entered data twice using Epi-Data software [18], with validation to minimize keystroke errors. Analysis relied on CIETmap open-source software [19], which provides a user-friendly interface with the R statistical programming language [20]. We examined associations with two dengue outcomes: self–reported cases of dengue illness in any household member in the last 12 months; and recent dengue infection among children aged 3–9 years, as evidenced by a doubling of specific dengue-specific IgG between the paired saliva samples. We examined associations between these outcomes and individual, household and community-level factors, first in bivariate analysis and then in multivariate analysis, using the Mantel-Haenszel procedure [21] with adjustment for clustering [22]. For multivariate analysis, we began with saturated models including variables associated with the outcome in bivariate analysis and performed step-wise deletion of the least significant association until all the remaining variables were significantly associated with the outcome at the 5% level. We tested for effect modification using Woolf’s ×^2^ test for heterogeneity [23]. We express associations as the odds ratio (OR) and cluster-adjusted 95% confidence intervals (95% CIca).

## Results

The field teams visited 12,399 homes in 90 clusters and collected information from 54,728 people; 81.2% (10,067/12,399) of respondents were women. Of 7048 children aged 3–9 years who provided the first saliva sample, 90.6% (6382/7048) provided a second sample. The loss to follow up was mainly due to families moving away from the area. Entomological inspections collected data from 45,013 water containers located inside or next to visited households.

### Characteristics of the communities and sample population

Table 1 shows characteristics of the communities. Few communities, especially in Costa Chica and Costa Grande regions, had a household drainage system or paved streets. Many had ditches or ravines where stagnant water could accumulate, and these were often blocked. Over half the communities had tap water inside the households (57/90). Most communities (68/90) had a water supply system: from a local source in 40, a municipal network in 28, and wells in 14. Only 43 had a regular water supply, the irregular supply forcing households to store water. The main sources of water when the piped supply failed were wells, water storage tanks and rivers. About half the communities had a waste collection system, though not always regular. In most of the communities, the key informants interviewed did not identify any organized community groups.

**Table 1:** Profile of the 90 baseline survey clusters, stratified by region

Features	Acapulco *n* = 30	Costa Grande *n* = 30	Costa Chica *n* = 30	Total *n* = 90
Drainage at households	16	7	8	31
Paved streets	11	2	1	14
Ditches or ravines present	27	28	27	82
Blocked ditches	16	8	12	36
Mean no. empty lots (range)	21.2 (0–85)	23.3 (0–96)	20.0 (0–96)	21.4 (0–96)
Uninhabited homes	18	21	9	48
Health center present	12	23	18	53
Tap water to households	19	17	21	57
Local water system	24	20	24	68
Regular water supply	7	16	20	43
Alternative source if supply fails— well or water storage tank	14	13	14	41
Garbage collection service	25	16	9	50
Regular garbage collection service	15	9	6	30
Garbage collection frequency: every three days	3	1	2	6
Adequate collection service	13	6	4	23
Organized groups^a^	8	5	7	20

^a^Taxi drivers, Alcoholics Anonymous, Oportunidades groups, religious/social/political groups

### Socio-demographic data

The mean number of people per household was 4.4 (range 1–24, SD 2.2). Some 53% (28,458/54,728) of household members were female, with a mean age of 28.4 years (range 0–90 years, SD 21.05). Nearly all households (90.9%; 11,265/ 12,396) spoke Spanish at home with a few reporting other languages, including *Ñomnda, Me’pha, Ñuu savi, Náhuatl,* and English. Similarly, 96% (11,900/ 12,396) of households reported Spanish as their preferred language for receiving health information. Some 40% (5041/ 12,399) of household heads had no education or did not finish primary school, 20.9% (2588/ 12,399) had completed primary education. The most frequent occupations of household heads were: farmer, 34.5% (4280/12,399); housewife, 9.5% (1184); merchant, 8.2% (1021); private employee, 7.2% (891); bricklayer or laborer, 6.8% (838); and driver or machine operator, 5.1% (637). Some 6.2% (772) were reported to be unemployed.

Nearly all the dwellings were used as households (94.5%; 11,720/ 12,399). About half the dwellings were of permanent construction (43.7%; 5424/ 12,399); 41.5% (5151) were semi-permanent, and a few were temporary (14.2%; 1762/12,399).

### Dengue illness

Across all three regions, 1.9% (1029/55,723) of household members were reported to have had dengue illness in the last 12 months. This ranged from 3.5% (663/19,174) in Acapulco region, through 1.5% (255/17,126) in Costa Grande, to 0.6% (111/18,423) in Costa Chica. One community in Acapulco region had a rate of 8.5% (55/648).

Self-reported dengue illness was more common among youth. The age groups with the highest rates were: 15–19 years, 2.9% (183/ 6419); 10–14 years, 2.6% (176/6.670); 5–9 years, 2.3% (131/5.677); 20–24 years, 2.2% (103/4599). Just over half the dengue illness cases were female (52.6%; 488/1029). Among children aged 3–9 years, 2.1% (163/7808) were reported to have had dengue illness in the last 12 months.

Table 2 shows bivariate analysis of factors potentially linked to self-reported dengue illness. Individuals were more likely to have had dengue illness if they were younger than 25 years, if they lived in an urban area, if they lived in Acapulco region, if their household was of permanent construction, if temephos had been placed in their household water containers within the last 3 months, if the household head had six years of education or more, if the household had less than five members, and if the household head was working (See Table 2).

**Table 2:** Bivariate analysis of associations with reported dengue illness in the last 12 months in 53,641 household members

Variable	Levels	With dengue		No dengue		OR	95%CIca
		Number	Percent	Number	Percent		
Age	<25 years	649	2.3	28,025	97.7	**1.56**	**1.32–1.85**
	≥25 years	380	1.5	25,612	98.5		
Sex	Male	488	1.9	25,747	98.1	0.98	0.86–1.11
	Female	541	1.9	27,889	98.1		
Area of residence	Urban	650	2.6	24,060	97.4	**2.1**	**1.46–3.04**
	Rural	379	1.3	29,581	98.7		
Region	Acapulco	663	3.5	18,481	96.5	**3.5**	**2.52–4.70**
	Costa Grande & Costa Chica	366	1.0	35,160	99.0		
Use of household	Business/ home business	63	2.4	2558	97.6	1.31	0.92–1.85
	Home	961	1.8	50,987	98.2		
Type of dwelling	Permanent	568	2.5	22,528	97.5	**1.73**	**1.34–2.23**
	Semi-permanent/temporary	449	1.4	30,820	98.6		
Temephos placed in water within last 3 m	Yes	689	2.2	31,013	97.8	**1.52**	**1.14–2.02**
	No	308	1.4	21,082	98.6		
Household use of anti-mosquito products	Yes	587	2.4	23,717	97.6	**1.69**	**1.40–2.04**
	No	435	1.4	29,717	98.6		
Education of household head	≥ 6 years of education	718	2.2	31,819	97.8	**1.64**	**1.32–2.05**
	<6 years or no education	294	1.4	21,367	98.6		
Working household head	Yes	902	2.0	44,950	98.0	**1.39**	**1.09–1.76**
	No	118	1.4	8148	98.6		
Language	Indigenous language	31	0.8	3843	99.2	**0.45**	**0.26–0.79**
	Spanish	790	1.8	44,276	98.2		
People per household	<5 people	483	2.4	19,887	97.6	**1.50**	**1.25–1.80**
	≥5 people	546	1.6	33,753	98.4		
Larvae-positive containers	Yes	138	1.6	8316	98.4	0.84	0.68–1.05
	No	891	1.9	45,325	98.1		
Pupae-positive containers	Yes	73	1.5	4746	98.5	0.79	0.61–1.01
	No	956	1.9	48,895	98.1		
Containers positive for larvae/pupae	Yes	141	1.6	8470	98.4	0.85	0.68–1.05
	No	888	1.9	45,171	98.1		

*OR* Odds ratio, *95%CIca* cluster adjusted 95% confidence interval

Figures in bold font indicate an association significant at the 5% level

Region was an effect modifier in the multivariate analysis of variables associated with dengue illness in the last year. We therefore constructed two models: one for Acapulco region, and another one for the Costa Grande and Costa Chica regions. Table 3 shows the final multivariate models. In the Acapulco region (Table 3a), factors associated with self-reported dengue illness in the last 12 months were: being younger than 25 years of age; having a household head who was working; having a household head with at least six years of education; having less than five people in the household; using anti-mosquito products in the household; and living in an urban site. In Costa Grande and Costa Chica (Table 3b), those more likely to report dengue illness in the last 12 months: were female; had a working household head; came from a household of less than 5 people; and came from a household that used anti-mosquito products.

**Table 3:** Final models of multivariate analysis of factors associated with reported dengue illness in the last 12 months in household members

Variable	Crude OR	adjusted OR	95%CIca
a) Acapulco region, *N* = 19,008, Clustered by site, *n* = 30			
Younger than 25 years	2.09	2.23	1.67–2.98
Working household head	1.57	1.40	1.03–1.89
Household head with ≥6 years of education	1.53	1.25	1.02–1.54
<5 people in household	1.45	1.59	1.25–2.02
Household uses anti-mosquito products	1.35	1.33	1.06–1.66
Urban residence	1.54	1.54	1.07–2.22
b) Costa Grande and Costa Chica regions; *N* = 35,461; Clustered by site, *n* = 60			
Female	1.27	1.28	1.04–1.57
Working household head	1.45	1.54	1.11–2.12
<5 people in household	1.50	1.53	1.14–2.05
Household uses anti-mosquito products	1.69	1.68	1.22–2.32

For Acapulco region, other variables included in initial model were: sex, type of dwelling, presence of temephos, language, and presence of containers with larvae/pupae

For Costa Grande and Costa Chica regions, other variables included in initial model were: age, area of residence, type of dwelling, presence of temephos, education of household head, language, and presence of containers with larvae/pupae

### Recent dengue infection among children

Across all three regions, 6.1% (392/6382) of children aged 3–9 years had evidence of recent dengue infection (based on quadrupling of dengue-specific IgG antibody levels between paired saliva samples); 9% (576/6382) based on trebling of IgG levels; and 17% (1083/6382) based on doubling of IgG levels. The rate of recent infection was higher in Acapulco than in Costa Grande and Costa Chica. (Table 4).

**Table 4:** Recent dengue infection in children aged 3–9 years, by region

Region	Percentage (proportion) with evidence of recent dengue infection					
	Quadrupling of IgG level	95%CI	Trebling of IgG level	95%CI	Doubling of IgG level	95% CI
Acapulco^a^	9.2% (184/1994)	8.0–10.6	12.1% (242/1994)	10.7–13.6	19.4% (387/1994)	17.7–21.2
Costa Grande^b^	5.2% (97/1869)	4.2–6.3	8.2% (153/1869)	7.0–9.5	16.5% (308/1869)	14.8–18.3
Costa Chica^b^	4.4% (111/2519)	3–6 – 5.3	7.2% (181/2519)	6.2–8.2	15.4% (388/2519)	14.0–16.9
Total	6.1% (392/6382)	5.6–6.8	9.0% (576/6382)	8.3–9.7	17.0% (1083/6382)	16.1–17.9

^a^Paired saliva samples in December 2009 and February 2010

^b^Paired saliva samples in May and November 2010

As shown in Table 5, the proportions of children with evidence of recent dengue infection were higher among children aged 3–4 years than among older children. The rates of reported dengue illness among children were much lower than the rates of serological dengue infection (Table 5). In contrast to the age pattern for serological dengue infection, the proportions of children reported to have had dengue illness in the last 12 months were higher among children aged 8 and 9 years than among younger children (Table 5).

**Table 5:** Recent dengue infection and reported dengue cases in last 12 months, in children aged 3–9 years, by age

Age (years)	Number of children	Recent dengue infections^a^	Recent dengue infections per 1000 children	Dengue illness in last 12 months	Dengue illness per 1000 children
3	742	93	125.3	9	12.1
4	894	92	102.9	15	16.8
5	824	63	76.5	14	17.0
6	953	82	86.0	18	18.9
7	921	82	89.0	16	17.4
8	1001	73	72.9	35	35.0
9	991	86	86.8	28	28.3
Total	6326	571	90.3	135	21.3

^a^Doubling of level of dengue specific IgG in paired saliva samples

Bivariate analysis found evidence of factors linked to recent infection: being aged 3–4 years; living in an urban area; and living in the Acapulco region (Table 6).

**Table 6:** Bivariate analysis of associations with recent dengue infection (doubling of IgG levels) among 6326 children aged 3–9 years

Variable	Level	With doubling of IgG		Without doubling of IgG		OR	95%CIca
		Number	Percent	Number	Percent		
Age	3–4 years	318	19.4	1636	80.6	**1.26**	**1.10–1.43**
	5–9 years	756	16.1	4690	83.9		
Sex	Male	568	17.6	3219	82.4	**1.10**	**0.96–1.26**
	Female	515	16.3	3161	83.7		
Area of residence	Urban	451	18.5	2442	81.5	**1.19**	**1.02–1.38**
	Rural	632	16.0	3940	84.0		
Region	Acapulco	387	19.4	1994	80.6	1.28	1.10–1.49
	Costa Grande & Costa Chica	696	15.9	4388	84.1		
Household use	Business/Home-business	51	21.1	242	78.9	1.32	0.99–1.78
	Home	1027	16.8	6123	83.2		
Type of dwelling	Permanent	401	17.2	2332	82.8	1.03	0.91–1.17
	Semi-permanent/ temporary	672	16.8	4011	83.2		
Temephos placed in water within last 3 m	Yes	602	17.5	2847	82.5	1.07	0.93–1.22
	No	458	16.5	2313	83.9		
Use of anti-mosquito products	Yes	500	18.1	2766	81.9	1.15	1.00–1.33
	No	579	16.1	3595	83.9		
Education	≥ 6 years of schooling	672	16.8	4010	83.2	0.96	0.84–1.08
	<6 years or no schooling	405	17.4	2327	82.6		
Working head of household	Yes	937	16.8	5563	83.2	0.96	0.77–1.19
	No	130	17.4	745	82.6		
People in household	<5 persons	267	16.9	1581	83.1	0.99	0.88–1.12
	≥5 persons	816	17.0	4801	83.0		
Larvae-positive containers	Yes	154	16.8	916	83.2	0.99	0.85–1.15
	No	929	17.0	5466	83.0		
Pupae-positive containers	Yes	89	17.1	521	82.9	1.01	0.80–1.27
	No	994	17.0	5861	83.0		
Larvae- and pupae-positive containers	Yes	159	17.1	931	82.9	1.01	0.86–1.18
	No	924	17.0	5451	83.0		

*OR* Odds ratio, *95%CIca* cluster adjusted 95% confidence interval

Figures in bold font indicate an associastion significant at the 5% level

Table 7 shows the final model from multivariate analysis of factors associated with recent dengue infection. Children aged 3–9 years old were more likely to have evidence of recent dengue infection if they were younger (aged 3–4 years), and if they lived in the Acapulco region.

**Table 7:** Final model of multivariate analysis of factors associated with recent dengue infection (doubling of IgG levels) among children 3–9 years of age *n* = 6326; Clustered by site, *n* = 90

Variable	OR	adjusted OR	95%CIca
3–4 years of age	1.26	1.25	1.10–1.43
Living in the Acapulco region	1.28	1.27	1.08–1.50

Other variable included in initial model was urban/rural residence

## Discussion

The survey which generated the findings reported here took place at the baseline of the Camino Verde trial [12, 13], before communities were randomised to intervention or control status. We used the findings about rates of dengue infection in different clusters to stratify the clusters prior to randomisation [12, 13]. The findings shed light on the conditions under which the Camino Verde intervention took place in the coastal regions of Guerrero, Mexico, and supported the management of the intervention there [24].

Evidence was the main tool for community mobilisation in Camino Verde and results from the baseline survey were an important part of that evidence. The field teams shared three kinds of results from the baseline communities: those from the collection of children’s saliva samples, those from the entomological inspections, and those from the household survey. Feedback of individual results from the saliva sample provided the opportunity for the *brigadistas* (community mobilisers) to approach every family in the community whose child provided two saliva samples during the baseline survey. In many cases it was the *brigadista*’s first contact with the household and it provided a natural opportunity to discuss dengue, the *Aedes aegypti* mosquito and its behavior [24]. Evidence from the entomological inspection, reported elsewhere [25], showed the types of containers where *Aedes aegypti* larvae and pupae were most concentrated, enabling the intervention team to prioritise these containers in their education efforts.

### Community organisation

As reported here, community key informants in the baseline survey in Mexico could not identify any organized community groups in three-quarters of the 90 sites (see Table 1). This was in sharp contrast with Nicaragua where most neighborhoods had recognized, active leadership closely allied with the Sandinista government. The Mexican intervention team had to spend considerable time and effort in search of suitable community leadership before the intervention could be fully launched [24].

### Community priorities

Levels of self-reported (that is, recognized) cases of dengue illness in the last 12 months were relatively low in the baseline survey in the 90 coastal clusters in Mexico: overall 1.9%, and lower in Costa Grande and Costa Chica. The rate of dengue illness in Acapulco was 2.3 times higher (3.5/1.5) than in Costa Grande, and almost six times higher (3.5/0.6) than in Costa Chica. In the baseline survey in Managua, Nicaragua, self-reported dengue cases were 24% higher and serological recent infection was double compared to Mexico [12]. The low levels of recognized clinical dengue infection in the trial population in Mexico, especially in areas outside the Acapulco region (and Acapulco City itself), meant that the *brigadistas* sometimes faced communities that had priorities other than dengue and were therefore less interested in getting involved in activities to reduce mosquitoes in their environment.

In the Mexican baseline study reported here, urban residence was associated with both self-reported dengue illness and serological recent dengue infection. Other authors have reported that dengue is a mainly urban phenomenon, with infection risk higher where there is disorganized urban growth, with lack of access to water, electricity, drainage, paved streets, education and health services [26]. Such conditions exist in Acapulco, which is not only a tourist attraction, but also a destination for many people who travel from within the State and the rest of Mexico looking for work. Despite the higher rate of dengue in urban sites in the baseline survey, the *Aedes aegypti* vector was present in every rural site [25] and frequent visits of rural dwellers to urban areas could put them at risk of contracting dengue and set off epidemic outbreaks in rural areas, as has been observed elsewhere [27].

### Self-reported dengue illness and dengue infection

The rates of recognized dengue illness in the baseline survey were well below the rates of dengue infection (as assessed by IgG levels in saliva) in children aged 3–9 years (see Table 5). It is recognized that many cases of dengue infection are not associated with clinical dengue illness [6]. The findings from the saliva examinations in children provided parents with evidence of how the infection could be affecting their children, even in the absence of obvious illness at the time.

Subclinical infections are important. They may contribute to continuing dengue transmission, as well as to the emergence of serious forms of the illness. According to illness surveillance reports, strands DENV1 and DENV2 were present in Guerrero in 2009, while DENV1 and DENV3 were identified in 2010 [1, 2]. Previous studies show that co-circulation of DENV strands increases the risk of epidemics and serious dengue cases [28].

### Age patterns

The incidence of self-reported dengue illness was higher among family members younger than 25 years. Other authors have reported a similar age gradient, and it may be that older adults have developed some level of immunity, having been exposed to all four serotypes—and been infected by at least one of them—over a longer period of time [29].

Among children aged 3–9 years, the age pattern for serological dengue showed higher rates among the youngest children (aged 3–4 years), while the age pattern for recognized dengue infection showed higher rates among the older children (aged 8–9 years) (see Table 5). The association between young age and serological evidence of recent dengue infection remained in multivariate analysis (Table 7). The higher rate of recognized dengue cases among the older children may be because older children are better at reporting symptoms; among younger children parents may confuse dengue fever with other health problems, such as a respiratory infection. Sharing the findings from the saliva samples helped to sensitize parents about the high rate of dengue infections among young children, which may go unrecognized even if they cause some non-specific symptoms.

### Other associations with self-reported dengue illness

Several factors in the baseline survey were associated with self-reported dengue illness among household members although not with serological dengue infection among children aged 3–9 years. In smaller (less than 5 people) households, households where the head was working, and households with a more educated head, the increased reporting of dengue cases may be because there is better recognition of cases of dengue illness. A study in Brazil reported an association between higher income and a higher risk of dengue haemorrhagic fever [30], and a study in Thailand reported a higher risk of dengue among more educated households [31].

### Anti-mosquito products

Use of anti-mosquito products was associated with self-reported cases of dengue illness in all three regions. Cause and effect in such associations are difficult to determine in cross-sectional studies. In the Mexican system of dengue prevention and control, health workers visit households with a recognized case of dengue fever to place temephos in the water and fumigate in the area [32]; this may sensitize households and lead them to increase their use of anti-mosquito products. Additional analysis of the baseline study highlighted high amounts being spent by households in anti-mosquito products [33], and the Camino Verde intervention emphasized non-chemical methods of vector control.

### Sex

Among children between 3 and 9 years old, boys were slightly more likely than girls to have evidence of recent dengue infection (Table 7), while in Costa Grande and Costa Chica self-reported dengue cases among all ages were more common among women (Table 3b). In the mainly rural Costa Grande and Costa Chica regions, customs are more traditional and women spend more time at home where they may be more exposed to the *Aedes aegypti* mosquito which prefers to breed in domestic settings. As it turned out, in Mexico women were much more active than men in community activities to reduce mosquitoes during the Camino Verde trial [34]; one reason for their greater involvement may be that they perceived themselves as more affected by dengue.

### Limitations

We identified cases of dengue on the basis of self-reports from the household respondent in each household. Such reports may not be fully reliable. Under-reporting of dengue cases may have been more marked in some households, as discussed above. We did not ask about the severity of symptoms, although we did collect information about whether cases had been admitted to hospital for treatment [35]. As with all cross-sectional analyses, we cannot interpret the associations we identified in our analysis as being causal.

## Conclusions

Findings from the baseline study of the Camino Verde trial confirmed and highlighted associations with dengue virus infection and with self-reported dengue illness. Discussion and dissemination of evidence from the baseline survey were integral to the intervention in the trial communities. The weakness of community leadership and the relatively low rates of self-reported dengue illness were challenges that the Mexican intervention team had to overcome. The higher occurrence of dengue illness among women in Costa Grande and Costa Chica noted in the baseline study may help explain why women participated more than men in achieving the successful outcome of the Camino Verde trial.

## Abbreviations

95% CI: 95% confidence interval
95% CIca: Cluster adjusted 95% confidence interval
DENV: Dengue virus
DF: Dengue fever
DHF: Dengue hemorrhagic fever
ELISA: Enzyme-linked Immunosorbent assay
IgG: Immunoglobulin G
OR: Odds ratio
